# Forensic Examination of Blood-Stains in the Tropics
^1^A dissertation for the M.B. degree taken *in absentia*, September, 1913.


**Published:** 1916-07

**Authors:** Francis Shingleton Smith

**Affiliations:** Captain, I.M.S.


### FORENSIC EXAMINATION OF BLOOD-STAINS IN THE TROPICS.[1]

BY THE LATE
FRANCIS SHINGLETON SMITH, B.A., M.B., B.C. Cantab.,
Captain, I.M.S.

---

In this essay I propose to describe in detail the examination of blood-stained articles and the determination of the nature of the blood as carried out in the Serological Laboratory in Calcutta. The Government of India instituted this laboratory for the performance of the bio-chemical tests for blood, and I have had the privilege of undergoing a course of instruction in this subject under Lieut.-Col. W. D. Sutherland, I.M.S., who is the organiser and director of the serological work. I would like here to acknowledge my indebtedness to him for permission to use the laboratory records for the purposes of this paper, and for the kind advice which he has always been so ready to give.

The blood-stained articles which came for examination from all parts of India and Burma may be classified as follows :—

1. Mud, earth, leaves and stones.
2. Swords and spears.
3. Knives of sorts : large sacrificial knives, Burmese dahs, Indian bill-hooks, sickles, daggers and pocket-knives.

[1] A dissertation for the M.B. degree taken *in absentia*, September, 1913.

4. Bamboos, " lathis," and small pieces of wood.
5. Clothes: dhotis, saris, kurtas.
6. Grains of sorts.

Owing to the climatic conditions, and the existence of a prolonged dry season preventing the washing away of blood-stains from the soil, the first class of articles is much more frequently dealt with than can be the case in temperate climates. Also the fact that natives of this country live in mud dwellings, even though these are frequently renovated inside with fresh coverings of cow-dung, made into a paste with water, chopped grass and earth, helps to swell the total number of these cases.

The cutting instruments are generally rusty, and the sacrificial knives are stained with goats' and sheep's blood, unless they have been recently cleansed.

Class 4 consists mainly of bamboos, though a " lathi " may be a stick of any sort.

Human blood is found on the bamboo in only a small proportion of cases; this may be accounted for by the presence of natural red markings on it, which to the ordinary mind suggest the probability of blood-stains, and also by the fact that red saliva ejected by betel chewers is frequently seen on staves as well as on many other articles for examination.

Clothes form the largest proportion of articles examined, and of these the " dhoti " is the commonest. This is a long, cotton loin-cloth for men, which also when required acts as a covering for the upper part of the body. The " sari " is a similar but more ornate and coloured garment for women, also serving to cover the head and the rest of the body. The " kurta," a shirt, is an additional garment for men.

There are several stains which mimic those of blood. They are red in colour and are generally quite easily distinguished at sight; the markings of the bamboo have already been mentioned, and similar markings occur on the stalks of cereals and of Jowari (Sorghum vulgare).

On clothes are found a variety of stains, among which that due to the saliva of betel chewers can usually be seen ; also marks of mud and filth of all kinds, for the ordinary peasant has not a large wardrobe, and wears his few garments until they are full of holes.

The stains are examined microscopically, spectroscopically, and when blood is detected by either of the first two methods of examination, bio-chemically.

### MICROSCOPIC EXAMINATION.

A small quantity of scrapings from the stained area in the case of earth, knives and bamboos, or a portion of the fabric in the case of clothes, is taken and allowed to soak in a drop of Vibert's solution (0.5 per cent. $HgCl_2$ solution in 2 per cent. $NaCl$), or in a drop of glycerine solution (one part in seven of water). It is then covered with a cover glass, and after standing for half an hour is tested and examined under low power, high power and oil immersion lenses. Mammalian blood-cells, and the oval granular nuclei of non-mammalian corpuscles can be detected ; in one case these were both found on a single grain of paddy (unhusked rice.)

### SPECTROSCOPIC EXAMINATION.

A small piece of the stained cloth is cut out, dipped into boiling water for five seconds to fix the stain, and is dried with blotting paper on a slide. A drop of potassium cyanide solution, 2 per cent., is dropped on to the specimen, and when there is blood we find small cherry-red areas under the low power. Two drops of an ammoniated solution of ammonium sulphide are added, and a cover slip is applied. The specimen is now ready for spectroscopic examination.

Instead of using boiling water to fix the stain, and then adding 2 per cent. cyanide, we find that the same result may be attained by adding a drop of saturated solution of potassium cyanide, which acts as a fixative.

The preparation, if thick, may be examined directly with the spectroscope, but it is usually necessary to examine it with the low and often with the high power of the microscope, after substituting for the eye-piece a spectroscope fitted with a scale of wave-length measurements. The specimen so prepared will, if blood is present, show the spectrum of cyanhæmochromogen, giving its characteristic bands.

In the case of earth, stones, sticks and knives a small drop of 2 per cent. potassium cyanide solution is placed on the blade of a clean scalpel, which is then used to scrape the stained area, and the stain solution so obtained is treated with the ammonium sulphide solution and then examined spectromicroscopically, as in the case of fabrics. If the examination with Vibert's solution is negative, and the cyanhæmochromogen bands are not seen, the case is returned as negative, "no blood detected." If otherwise, we proceed to carry out the precipitin reaction.

The guaiacum test (Van Deen's) is not applied, even as a negative test. The reasons for this are set forth in Lieut.-Col. Sutherland's *Blood-Stains, their Detection and the Determination of their Source* (London, 1907).

### THE PRECIPITIN REACTION.

This depends upon the principle that if an animal of species A receives injections (preferably intravenous) of the serum of an animal of a not too closely related species B, its serum will, in time, develop the power of causing a precipitate to form, when it is brought into contact with a high dilution of the serum of species B, but not when brought into contact with a high dilution of serum of other species, save of those very closely related to B.

Now, if a fowl receives injection of the serum of a horse, that fowl's serum will, after a time, have the power of causing a precipitate to form when it is brought into contact with a high dilution of horse's serum, and also ass's serum ; but no such reaction will occur if it is brought into contact with a high dilution of the serum of the goat, the cat, the camel, a man, etc.

The reaction appears to be due to a change of the electric potential of the albuminous molecules of the diluted serum. These molecules, being colloid, are in a state of suspension in the dilution. If this be true, then, if the electric potential be that of the molecules of the treated animal's serum, they will keep separate from these, and no precipitum will b formed. If, on the contrary, their potential be the opposite of that of the molecules of the treated animal's serum, they will join these and thus cause the formation of a precipitum.

As a matter of fact we find that, in adding anti-serum to a dilution of serum, if a zone of precipitate has formed, and an excess of either serum dilution, or of treated animal's serum be added, this precipitate *dissolves* ; the excess has caused all the molecules present to take on the electric potential of the matter in excess, and thus being all of one and the same potential, the molecules remain separate from each other, hence this explanation of precipitation is preferable to that which is in part based on surface-tension.

Before the test can be applied we require, firstly, normal serum of various species for injection into the animals that are to elaborate our antisera. The blood, from whatever source and however obtained, is allowed to clot and the clear serum is at once pipetted off into sterile bottles. It is then inactivated by being heated to 56° C. for half an hour, and stored in the freezing chamber till required.

Owing to the sanctity of the ox in the eye of the Hindu, we use buffalo serum ; the anti-serum obtained from the animal injected with this acts strongly with the sera of the buffalo and ox, and also weakly with those of the goat and sheep, so that the differentiation of buffalo and ox antigen by anti-bubaline serum is only one of degree, depending on the difference in time between the appearances of the reactions and on the quantity of precipitum. The reaction of such anti-serum with goat or sheep antigen shows the same difference in degree carried a step further, so to speak.

We use no simian blood for injections, firstly, because the ordinary Lungoor (Semnopithecus entellus) is regarded as sacred, and it has not been argued in a court of law that the blood in question reported as human by the laboratory might be simian; secondly, according to the experiments of Sutherland, a reasonably strong anti-human serum should differentiate sufficiently between the blood of man and monkey within the time limit of twenty minutes. He examined blood-stains due to the blood of a number of varieties of the apes of the old world, and he states as follows :—

"It was found that even in the orang's blood-stain the reaction obtained was not human as it was not visible till after the expiration of twenty minutes, whilst the human blood-stain extracts and dilute sera in each case reacted well before five minutes had elapsed. These experiments were repeated over and over again, etc." and in no case was human reaction obtained with the extract of a simian blood-stain.

For the elaboration of anti-sera, rabbits are difficult to obtain and are prone to disease, so that fowls are used, as they yield satisfactory anti-sera. Two injections of the antigenic blood serum are given into the axillary veins, the doses are 4 c.c. and 8 c.c. at three days intervals. Fourteen days later, after a twenty-four hours' cessation of feeding, the fowls are bled into sterile flasks and the blood allowed to clot. When this has occurred, the serum is pipetted off into sterile bottles and stored in an ice chest.

In the laboratory in Calcutta no antiseptics are used for preservation purposes, the anti-serum being kept frozen and in the dark. Sutherland[1] found that anti-sera exposed to light and warmth at room temperature became absolutely inert in two or three days in Calcutta.

### THE PRECIPITIN TEST IN PRACTICE.

The anti-human fowl serum is taken from the ice chest and allowed to remain in the dark at room temperature in order to thaw gradually. It is found that if suddenly warmed such anti-sera are unreliable and may give reactions with the dilutions of all the normal sera, but that if again tested after an hour or two the same anti-sera have regained their specificity. Having thawed it sufficiently a sterile capsule is filled with the anti-serum, labelled and placed in the cupboard till required. The bottle containing the stock solution is replaced in the freezing chamber.

The anti-serum has now to be tested as to its potency and specificity. This is done every day that it is used, and the technique is as follows: Tubes containing normal sera of the domestic animals and man are taken. The sera we use are human, hircine, bubaline, canine, feline and equine. Dilution of one in a thousand of 0.85 per cent. salt solution are made from each of these sera. Of these dilutions at most two cubic centimetres are poured into the pointed tubes which we use for the test, and one tube of salt solution alone is put up. Two drops of anti-human fowl serum from the capsule are allowed to run down the side of the tube, which is held nearly horizontal and has been moistened with the serum dilution it contains. The slightly coloured anti-serum can be seen descending to the bottom of the tube, being heavier than the normal serum, and the tubes are placed in the rack. If, as is finally the case, the tube containing the human serum shows a marked reaction in about two minutes, and there is no reaction visible in the other tubes for more than twenty minutes after the addition of the anti-serum, it is considered to be potent and specific, and is used for testing the extracts of blood-stains sent for examination. The reaction consists in the formation of a cloudiness at the junction of the serum and anti-serum, best seen by examining the tube contents against a black background. For this purpose we use a piece of cardboard covered with black cloth. Two drops of anti-sera are used in order that there may be a little clear fluid below the ring of precipitum, thus making the cloudiness more apparent by contrast above and below.

Having proved that the anti-serum is potent and specific, we proceed to prepare our stain extracts for examination by its means. These extracts are made by adding a small quantity of physiological salt solution, 0.85 per cent., to the fragment of cloth, scrapings from a weapon or missile, portion of blood-stained earth, etc., which has been proved to be stained with blood as already detailed. If the blood-stain gives an extract readily, this is at once filtered in order to obtain it as clear as possible before small extraneous particles have become suspended. It often happens that owing to the hot climate and the age of the stain it does not readily become extracted ; in such cases the addition of a few drops of a weak solution of cyanide of potassium to the tube contents will hasten extraction. We owe this manœuvre to Ziemke.[2] One drop of a 2 per cent. solution if added to one c.c. of salt solution will have the desired effect. The great desideratum is to obtain as concentrated a solution as possible in the test tube at this stage.

If the extract be difficult to clear by filtration, it should also be centrifuged and then again filtered ; very few extracts save those of some earths resist these means of clarifying.

When cyanide of potassium has been used, the alkalinity of the extract must be neutralised by the addition of a drop or two of a weak solution of tartaric acid. The extract is then diluted until it corresponds to a $\frac{1}{1000}$ dilution of serum, as is shown by the foam test. The dilutions are then treated with litmus to ensure the absence of acidity or of excessive alkalinity, and at most two cubic centimetres are formed into a second series of tubes, pointed and numbered according to the numbers on the test tubes containing the stain extracts. To the contents of each tube are now added two drops of anti-human serum, which has already had its potency and specificity confirmed. Every two or three minutes the tubes are examined, and only those stains whose dilute extracts give a reaction within the time limit of twenty minutes are entered as being due to human blood, *i.e.* they contain human albumin as proved by the precipitin test.

It is necessary to fix a time-limit in order to prevent the "mammalian reaction" from vitiating the results. As shown by Nuttall, the test is not absolutely specific: an anti-human serum that reacts well with its homologous serum will also react with other mammalian bloods if a long enough time is allowed, consequently the time-limit of twenty minutes has been adopted here, as in most laboratories.

In testing the specificity of an anti-serum, it sometimes happens that a reaction occurs with a dilution of a serum other than that used as the antigen. We have had one horse serum which habitually reacted with anti-human fowl serum; on inquiry it was found that the horse from which the serum had come was diseased.

One or two specimens of anti-human sera gave an appreciable reaction with feline and other non-human sera in dilution within the prescribed time-limit. Dr. G. E. Mitra carried out a series of experiments (hitherto unpublished) whose results go far to show that by diluting such an anti-serum—if it be highly potent for its antigen—with normal fowl serum, it can be rendered useful for forensic work by having its potency retained in sufficient degree, whilst its specificity has been increased so that no untoward reaction will occur for half an hour.

The side action of an anti-serum with a serum closely related to the homologous serum has been overcome by Weichardt (quoted by Sutherland), who used the exhaustion method; but Sutherland,[1] working in India, found that by removing the side action of an antiovine serum for goat serum he had also destroyed its action in its homologous serum.

In this laboratory an anti-serum which shows a mammalian reaction within twenty minutes, or a side action on the serum of a closely-related species, is not used for medico-legal purposes, though it is probable that soon such anti-sera corrected by Mitra's method will come into use.

The possible sources of error in the reaction are as follows :—

1. Presence of mineral acid, or strong alkali. The tubes and extracts must be tested for acid with litmus, and neutralised if necessary with sodium bicarbonate. If potassium cyanide has been used in the extraction of the stain it must be neutralised by tartaric acid.

2. Dirty tubes. The tubes should be examined against a dark background, and any that are not absolutely clean, or are frosted, should be discarded.

3. Cloudy anti-sera. No anti-serum that is not clear should be used; it does not matter if the anti-serum has a slight red colour from dissolved hæmoglobin, in fact this helps to fix the level at which the reaction would occur.

4. Extracts cloudy. Stain extracts must also be clear. How this may be brought about has been described.

5. Too rapid thawing of anti-sera that have been kept frozen. The thawing of the anti-serum when taken from the freezing chamber must be gradual. If this precaution is not observed, reactions are liable to occur with all the antigens when the anti-serum's specificity is tested.

6. Too little anti-serum used for the test. At least two drops of anti-serum should be added to each tube of stain extract, otherwise the reaction will occur at the very bottom of the tube, and thus will not be so well defined as it is when there is some clear fluid below.

7. Omission to test the anti-serum before use in a forensic test, as either its potency or specificity, or both, may have undergone a change by keeping.

8. Unsuitable dilution of stain extract. A precipitum is soluble in excess of anti-serum or of antigen.

Neither putrefaction nor age affects the test to any great extent.

The following specimens were examined by me :—

| | *Article.* | *Date of Stain.* | *Date of Exam.* | *Result.* |
|---|---|---|---|---|
| 1. | Clear stain on cloth . . | 1.7.09 | 14.5.13 | Human blood. |
| 2. | do. do. . . | do. | do. | do. |
| 3. | Red stain on cloth . . | 18.9.09 | do. | do. |
| 4. | Black powder . . . . | 17.9.09 | do. | Spectro.-negative |
| 5. | Red stain on cloth . . | 22.1.13 | do. | Human blood. |

All the positive reactions occurred within five minutes. The anti-serum reached with $\frac{1}{1000}$ dilution of its antigen in three minutes.

Uhlenhuth[3] failed to obtain reaction with old Egyptian mummies, but with mummies of 66 years and under he was successful in every case.

The general results were as follows. Of 107 specimens of earth, cow-dung, stones and grass examined 72 were found to contain blood, and of these in 44 the blood was proved by means of the precipitin reaction to be of human origin.

BIBLIOGRAPHY.

[1] Sutherland, W. D., *The Applicability to Medico-legal Practice in India of the Bio-chemical Tests for the Origin of Blood-Stains.* Sci. Mem. Series, No. 39. Calcutta, 1910.

[2] Ziemke, E., "Zur Untersssheidung von Menschen. und Tierblut mit hilfe eines spezifischen Serums," *Deutsche Med. Woch.*, 1901.

[3] Uhlenhuth, P., "Ueber die Bestimung der Herkunft von Mammien material mit Hilfe specifischer Sera," *Deutsche Med. Woch.*, 1905.

---

